# Prion strains in mammals: Different conformations leading to disease

**DOI:** 10.1371/journal.ppat.1006323

**Published:** 2017-07-06

**Authors:** Rodrigo Morales

**Affiliations:** Mitchell Center for Alzheimer’s Disease and Related Brain Disorders, Department of Neurology, The University of Texas Health Science Center at Houston, Houston, Texas, United States of America; Washington University School of Medicine, UNITED STATES

## Strain variation in prion diseases

Prion diseases are neurodegenerative disorders affecting mammals with a diverse etiology. Although rare, most of the cases occur spontaneously in humans, with a minority being inherited or acquired by infection. Prion disease in ruminants such as sheep, goat, and deer are relatively frequent and likely feed borne [1] or environmentally transmitted [2]. The confirmed zoonotic potential of bovine spongiform encephalopathy (BSE) and the still unknown consequences of other animal prionopathies to humans have placed these diseases in the spotlight.

“Prions” refers to proteinaceous infectious particles. This concept was originally defined after the pathological properties of the disease-associated prion protein (also termed PrP^Sc^). These unique infectious agents exist in a wide variety of “strains.” Prion strain variation was first suggested in 1961 by Pattison and Millson, who identified different phenotypes in experimentally infected goats [3]. This concept was later proven in inbred laboratory rodents, which also showed prion disease-specific phenotypes, brain-lesion profiles, and incubation periods [4]. The existence of prion strains was originally difficult to rationalize with the idea that the causative agent in these diseases was composed only by misfolded proteins, particularly considering that, in most cases, strain variation was documented within the same animal species (expressing a single cellular prion protein [PrP^C^] sequence). Current evidence suggests that the main difference between prion strains lies in the different conformational arrangements that PrP^Sc^ acquires [5]. These infectious particles “transmit” their particular conformational motifs to the normally folded proteins expressed in the host, leading to specific disease features.

## How can prion strains be distinguished?

**Fig 1:**
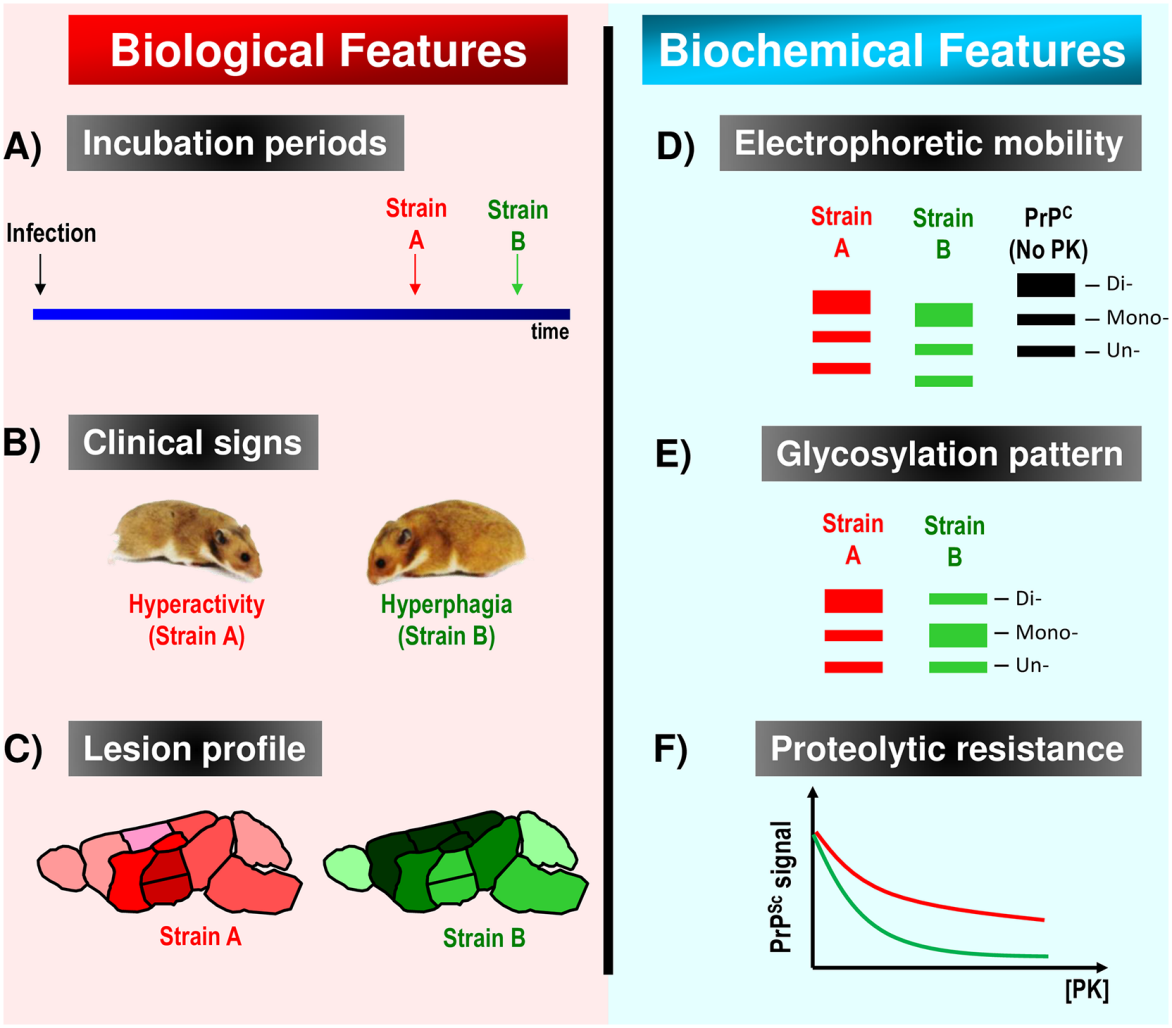
Differential biological and biochemical features used to distinguish between prion strains. **(A)** Incubation periods to disease are one of main features allowing differentiation between prion isolates. Time differences between the incubation periods of 2 prion strains may vary depending on the route of administration. **(B)** Different clinical manifestations may also help to discriminate between different prion agents. This property has been very useful in some species (including goats and hamsters) but inefficient in others (such as mice). For example, signs as dissimilar as hyperactivity and hyperphagia can be observed within a single animal species (in this case, Syrian hamsters). **(C)** Prion isolates are known to induce spongiform degeneration in different areas of the brain in a strain-specific manner. Recently, similar profiles have been adapted for the disease-associated prion protein (PrP^Sc^) deposition as well. In this graphic example, darker color in certain brain areas represents more severe damage. **(D)** At the molecular level, the partial proteolytic resistance of PrP^Sc^ lies in its C-terminal region. Strain-specific prion folding is thought to protect different lengths of the polypeptide chain from proteases, leading to different electrophoretic mobilities. In this western blot model, no–Proteinase K treated cellular prion protein (PrP^C^) is shown as comparison (black). **(E)** The ability of certain prion arrangements to recruit specific PrP glycoforms also helps with easy strain characterization by western blots. **(F)** Resistance to proteolytic degradation by increasing the concentration of proteases is commonly used to investigate the identity of putatively different prion isolates.

In addition to clinical signs, methods have been designed to confirm the identity of different prion agents. These methods are based on the particular biochemical properties of each prion variant or the pathological features they generate in the host. A description of the most widely used techniques to differentiate prion strains is summarized in Fig 1. Distinguishing strains by these criteria has become routine in rodent models, while identification of strains in natural hosts is more challenging and less reliable. Moreover, the overall ability to identify unique strains is limited because of the inability of current techniques to determine the detailed 3-dimensional features of each PrP^Sc^ variant.

Incubation periods to disease are perhaps the most-used observations to determine the identity of a prion agent. This is obviously used in experimental settings because only controlled laboratory conditions allow the time lapsing between infection and clinical manifestations to be measured. Another disease feature allowing prion strain differentiation involves the specific patterns of spongiform degeneration in the brain that are generated when a single animal species is infected with different PrP^Sc^ isolates. In 1968, a method to assess the extent of spongiform degeneration of a mouse-adapted scrapie isolate in 9 brain regions was described [6]. The ability of this procedure to discriminate between different prion strains is still widely used today. Consequently, damage at different brain regions by different prion strains provides a rationale to explain the distinct clinical signs routinely observed in laboratory settings. This is well exemplified on Syrian hamster prion diseases, in which clinical signs as dissimilar as hyperactivity (Hyper [HY] isolate [7]), drowsiness (Drowsy [DY] isolate [7]), and hyperphagia (SSLOW isolate [8]) can be found.

Several techniques have been developed to distinguish PrP^Sc^ variants at the biochemical level. Among them, electrophoretic mobility after proteinase K (PK) digestion is the most commonly used method because is easy to visualize while providing indirect evidence of conformational changes. The partial resistance of prions to proteolytic degradation lies within the C-terminal region. However, the length of the PK-resistant segment varies depending on the prion strain analyzed. This can be easily distinguished in western blots [5]. However, it is not uncommon that phenotypically different prion strains result in the same electrophoretic mobility. Another feature to differentiate among prion strains involves their glycosylation profiles. The prion protein has two putative glycosylation sites, allowing it to exist in di-, mono-, or unglycosylated forms. Interestingly, each PrP^Sc^ variant favors specific proportions of PrP glycoforms, facilitating their characterization [5]. Other biochemical techniques used to differentiate among prion strains rely on their extent of proteolytic degradation or denaturation when increasing concentrations of PK or chaotropic agents are used. Advances on other biophysical methods to analyze protein structure continue and are expected to improve resolution necessary for better understanding strain-specific differences among PrP^Sc^ strains [9].

## Implications of strain variation on interspecies prion transmissions

Prion strain variation occurs in virtually all susceptible animal species and is independent of the disease’s etiology. As an example, different strains of human PrP^Sc^ can be generated spontaneously or by interspecies infection. A major source for strain variation in experimentally induced prion diseases is the interspecies transmission of the agent. Once interspecies transmission of prions is overcome, new prion strains commonly emerge [5]. The isolates generated upon a first passage are likely a mixture of different PrP^Sc^ types [10], containing particles with similar properties as the original source. Nevertheless, stabilization of the infectious materials by serial infectivity bioassays may generate completely different syndromes [7], but this phenomenon is entirely case specific.

Likewise, each prion isolate demonstrates independent potentials for interspecies infection. For instance, transmissible mink encephalopathy (TME) prions readily infect raccoons but are completely innocuous in wild-type mice. Although the primary sequence of host’s PrP^C^ is largely responsible for this phenomenon, similar events can be found when prion strains from a single species (likely having different conformational features) are tested in a second one. When hamster prion isolates DY and HY (both originated from TME [7]) are used to infect minks, DY prions do so with relatively short incubation periods and complete attack rates, whereas the HY isolate demonstrates a strong transmission barrier [7]. This example proves that the generation of new strains due to interspecies transmission plays a significant role for their potential to infect other animal species. It remains important to emphasize that the transmission of certain prion strains (such as BSE) to “intermediate” animal species may result in new prion isolates with unprecedented zoonotic potentials.

## Additional sources of strain variation in prion diseases

The role of polymorphisms on prion strain variation has also been described for several animal species. Sporadic prion diseases in humans can be caused by different PrP^Sc^ types, and polymorphisms at amino acid 129 (methionine or valine) in the prion protein are important for strain diversity in this species. However, at least three PrP^Sc^ strains have been identified just with methionine at this position. The effect of polymorphisms on prion strain variation has also been described in other species, such as sheep and goats [11]. The existence of polymorphic changes in the mouse prion protein gene (*prnp*) allowed modeling the extent of this feature over disease transmission [12]. Importantly, interpolymorphic transmission of prions appears to act as interspecies infections in terms of the barriers to replicate prions from one group into a second one.

An additional experimental source of strain variation involves misfolded arrangements of synthetic PrP that are capable to induce disease upon inoculation. Examples of these are SSLOW and MoSP1 strains in hamsters and mice, respectively [8,13]. Both have been shown to generate unique phenotypes, clearly different than other prion strains previously established in these species.

The events favoring the selection of a particular strain are not clear. However, it is suggested that a particular prion agent emerges either from a pool of different structural motifs from which some are selected by still unidentified factors [10] or evolves due to changes in the replication environment [14]. Experimentally, prion selection can be achieved by limited dilution after interspecies infections [7] and the use of chemical compounds [15]. Remarkably, some reports have shown that different PrP^Sc^ types (as judged by their electrophoretic profiles) can be generated in brain and peripheral tissues [16]. Future experiments would define whether they truly correspond to different prion isolates or are just a consequence of the environment in which they are propagated.

## The strain phenomenon beyond the realm of prion diseases

Several other diseases associated with protein misfolding have been shown to progress following prion-like mechanisms. Thus, prion-like properties have been tested for non-PrP^Sc^ protein aggregates. Early approaches using purified amyloid-β (Aβ) peptides showed that conformationally different aggregates can be serially propagated [17]. Strain-specific propagation in cell cultures was later shown for tau and α-synuclein fibrils [18,19], suggesting that conformational strains may naturally exist for other protein aggregates besides PrP^Sc^. Recent data from patients and animal models strongly favor this idea. For Aβ, the fate of the aggregates generated in mice by exogenous “seeding” depends on both the seeds administered and the host [20]. Moreover, conformationally different aggregates from synthetic fibrils and deposits isolated from patients were propagated in the brains of transgenic mice expressing human Aβ [21,22]. Astonishingly, when a panel of brain tissue from human tauopathies (showing morphologically different aggregates) was injected in mice expressing human tau protein, seeded aggregates propagated similar lesions as compared to the donors [23]. Together, one can suggest that proteinaceous conformational strains exist and importantly participate in the pathophysiology of several prevalent protein-misfolding disorders.
